# 3D Ultrastructural Visualization of Mitosis Fidelity in Human Cells Using Serial Block Face Scanning Electron Microscopy (SBF-SEM)

**DOI:** 10.21769/BioProtoc.4708

**Published:** 2023-07-05

**Authors:** Nuria Ferrandiz, Stephen J. Royle

**Affiliations:** Centre for Mechanochemical Cell Biology and Division of Biomedical Sciences, Warwick Medical School, University of Warwick, Gibbet Hill Road, Coventry, CV4 7AL, UK

**Keywords:** Chromosome missegregation, Mitosis, Endoplasmic reticulum, Volume EM, Microscopy, Scanning electron microscopy

## Abstract

Errors in chromosome segregation during mitosis lead to chromosome instability, resulting in an unbalanced number of chromosomes in the daughter cells. Light microscopy has been used extensively to study chromosome missegregation by visualizing errors of the mitotic spindle. However, less attention has been paid to understanding spindle function in the broader context of intracellular structures and organelles during mitosis. Here, we outline a protocol to visualize chromosomes and endomembranes in mitosis, combining light microscopy and 3D volume electron microscopy, serial block-face scanning electron microscopy (SBF-SEM). SBF-SEM provides high-resolution imaging of large volumes and subcellular structures, followed by image analysis and 3D reconstruction. This protocol allows scientists to visualize the whole subcellular context of the spindle during mitosis.

## Background

Cell division is essential for living organisms, as it is important for growth, repair, development, and reproduction. Accurate chromosome segregation during mitosis is essential to maintain genomic stability in cell division. Entry into mitosis involves a dramatic, large scale cellular reorganization. After the nuclear envelope breaks down, the chromosomes congress at the cell equator. The mitotic spindle, a complex subcellular machine, coordinates this congression and then the accurate segregation of sister chromatids to the two daughter cells. The combination of light and electron microscopy (EM), together with computer-based 3D reconstruction, shows that the mitotic spindle is localized in an exclusion zone, which is free of endomembranes: nuclear envelope and Golgi remnants, endoplasmic reticulum (ER), and vesicles (Puhka et al., 2007; Lu et al., 2009; Puhka et al., 2012; Champion et al., 2017; Nixon et al., 2017). Beyond the exclusion zone, the endomembranes are densely packed and mitochondria are randomly distributed. Recently, we described how chromosomes that do not congress to the cell equator can lie beyond the exclusion zone and become ensheathed by endomembranes, constituting a risk factor for chromosome instability (Ferrandiz et al., 2022). Our aim here is to provide a detailed step-by-step protocol to visualize, at the ultrastructural level, these chromosomes that become ensheathed by endomembranes. We use light microscopy to locate the cell of interest and then semi-automated large volume EM method (serial block-face scanning electron microscopy, SBF-EM) and 3D reconstruction to visualize the mitotic cell. The protocol will be useful for other applications where the total endomembrane content of mitotic cells, or indeed non-dividing cells, needs to be visualized.

## Materials and reagents

**Cell culture**

1. DMEM/F-12 Ham (Merck Life Science, catalog number: D6421-6)
2. Fetal bovine serum (FBS) (Sigma, catalog number: F7524)
3. Trypsin-EDTA solution (Sigma, catalog number: T3924)
4. l-glutamine (Sigma, catalog number: G7513)
5. Penicillin/streptomycin (Gibco, catalog number: 15140-122)
6. Sodium bicarbonate (NaHCO_3_) (Sigma, catalog number: D8662)
7. MatTek gridded glass-bottom culture dishes (MatTek Corporation, catalog number: P35G-1.5-14-CGRD)
8. Fugene HD transfection reagent (Promega, catalog number: E2312)
9. OptiMEM (Gibco, catalog number: 10149832)
10. GSK923295 (Selleckchem, catalog number: s7090)
11. Thymidine (Sigma, catalog number: T1895)
12. RO-3306 (Sigma-Aldrich, catalog number: SML0569)
13. SiR-DNA (Spirochrome, catalog number: SC007)
14. Poly-L-Lysine (Sigma-Aldrich, catalog number: P8920)
15. Concanavalin A (Sigma-Aldrich, catalog number: C2010)

**Sample processing**

1. Sodium phosphate dibasic (Na_2_HPO_4_) (Sigma, catalog number: S0876)
2. Sodium phosphate monobasic (Na HPO4) (Sigma, catalog number: S0751)
3. Potassium hydroxide (KOH) (Sigma, catalog number: 221473)
4. Glutaraldehyde 25% (Agar, catalog number: R1011)
5. Paraformaldehyde 16% (Thermo Fisher, catalog number: 28908)
6. Tannic acid, low molecular weight (Electronic Microscopy Science, catalog number: 21710)
7. OsO_4_ (TABB, catalog number: O014)
8. Potassium ferrocyanide (Sigma, catalog number: 14459-95-1)
9. Thiocarbohydrazide (TABB, catalog number: T009)
10. Uranyl acetate (UA) (TAAB, catalog number: U008)
11. Methanol anhydrous (Sigma, catalog number: 322415)
12. Lead nitrate (TAAB, catalog number: L019)
13. l-Aspartic acid (Sigma, catalog number: A9256)
14. Ethanol molecular grade (Sigma, catalog number: 51976)
15. Agar 100 premix kit-Hard (Agar Scientific, catalog number: R1140)
16. Silver conductive paste (TAAB, catalog number: S384)
17. Gold/Palladium (80/20%) 60 mm × 0.1 mm (Quorum technologies, catalog number: SC500-314B)
18. 0.1 M phosphate buffered (PB) saline (see Recipes)
19. Fixation buffer (see Recipes)
20. 2% reduced osmium (see Recipes)
21. 1% thiocarbohydrazide (TCH) solution (see Recipes)
22. 1% uranyl acetate (UA) (see Recipes)
23. Lead aspartate solution (see Recipes)
24. Agar 100 premix kit (see Recipes)

## Equipment

1. Nikon CSU-W1 spinning disc confocal system with SoRa upgrade (Yokogawa). Used with a Nikon, 20×/0.50 objective (Nikon) with optional 2.3× intermediate magnification or 60×, 1.40 NA, oil, Plan Apo VC objective (Nikon), and 95B Prime camera (Photometrics). The system has CSU-W1 (Yokogawa) spinning disk unit with 50 μm and SoRa disks (SoRa disk used), Nikon Perfect Focus autofocus, Okolab microscope incubator, Nikon motorized XY stage, and Nikon 200 μm z-piezo. Excitation was via 405, 488, 561, and 638 nm lasers with 405/488/561/640 nm dichroic and Blue, 446/60; Green, 525/50; Red, 600/52; FRed, 708/75 emission filters
2. Ultramicrotome (Leica Microsystems, EM UC7)
3. Gatan 3 View system installed on an FEI Quant 250 ESEM, with digital micrograph software (Gatan)

## Software

1. NiS Elements (Nikon)
2. Digital micrograph software (Gatan)
3. Open-source programs for analyzing images such as:
  - Fiji (https://fiji.sc) (version 2.9.0)
  - Microscopy Image Browser (http://mib.helsinki.fi) (version 2.60)
  - IMOD (https://bio3d.colorado.edu/imod/) (version 4.10.49)

## Procedure

- **Cell culture (Timing: 2–4 d)**
  1. This protocol can be adapted to most mammalian cell lines; we have imaged HeLa, RPE1, and HCT116 cell lines using this protocol. Here, RPE1 GFP-Sec61β cells were maintained in DMEM/F-12 Ham supplemented with 10% FBS, 2 mM l-glutamine, 100 U/mL penicillin/streptomycin, and 0.26% NaHCO_3_ in a humidified incubator at 37 °C and 5% CO_2_. Seed the cells 24 h before starting the experiment (see Note 1). Seed approximately 90,000 RPE-1 GFP-Sec6β cells onto MatTek gridded glass-bottom culture dishes (see Note 2) once they are detached using trypsin-EDTA solution. The alphanumeric photoetched grid provides a reference for tracking the same cell throughout the workflow. The degree of confluency, ideally 60%, on the day of imaging, is important to achieve the aim. We require as many cells as possible at the correct mitotic stage determined by the fluorescent tags. However, too many cells can obscure the photoetched grid and make correlation difficult.
*Note 1: The specific media and additives mentioned in this section are for RPE1 cells. If a different cell line is being used, please use the appropriate media and additives for that cell line.*
*Note 2: If transfection is required, approximately 25,000 RPE1 GFP-Sec61b cells need to be seeded 48 h before starting the experiment. RPE1 cells are transfected using Fugene (3:1); this ratio is a guide, depending on the plasmid size and cell type. Add 3 μL of Fugene to 100 μL of OptiMEM, incubate for 5 min, and add a total of 1 μg of purified plasmid DNA to the mixture of OptiMEM-Fugene. Incubate for 20 min at room temperature (RT), then add to the cells in the MatTek dish containing whole DMEM/F-12 Ham, and leave for 12–16 h. Typically, this results in around 20%–30% transfection efficiency. The following morning, wash off the transfection media and replace with full DMEM/F-12 Ham. Cells are then imaged by light microscopy 48 h later.*
  2. Antimitotic drug treatment to induce chromosome misalignment (see Note 3): Our protocol uses 150 nM GSK923295, a CENP-E inhibitor, for 3 h. Wash off the drug 1 h before imaging to allow cells to enter mitosis. Typically, around 30% of cells are at metaphase in this protocol.
*Note 3: OPTIONAL: Coating the glass coverslip with poly-l-lysine or concanavalin reduces the risk of mitotic cells washing away during imaging and sample processing.*
*Note 4: OPTIONAL: Mitosis is a rare event, occurring for 1%–8% of the cell cycle; if a specific stage needs to be visualized, this reduces the chance that a cell of interest can be readily found. The way to increase the chance to capture the desired cell is to synchronize them or use drugs to arrest them in early mitosis. Our current protocol for chemical synchronization uses a 2.5 mM thymidine block for 16 h, release for 6 h, and incubation in 9 μM RO-3306 for 16 h. After that time, RO-3306 is washed off for 45 min before imaging. This results in 30% of the cells being at metaphase.*
  3. To dye chromosomes in live-cell imaging, incubate cells with 0.5 μM SiR-DNA for 30 min before imaging commences.
- **Light microscopy**
  1. Keep cells in full DMEM/F-12 Ham media during imaging.
  2. The microscope used in our experiments is a Nikon CSU-W1 spinning disc confocal system with SoRa upgrade (Yokogawa). Sora disk is used with Nikon 20×/0.5 with optional 2.3× intermediate magnification or 60×, 1.40 NA, oil, Plan Apo VC objective, and 95B Prime camera (Photometrics). The system has the Okolab microscope incubator used for imaging at 37 °C and 5% CO_2_. Acquisition and image capture was via NiS Elements (Nikon). A widefield microscope would also be sufficient to visualize the cells.
  3. Select mitotic cells of interest by light microscopy (desired stage of mitosis, moderate fluorescent protein expression, etc.). For our experiment, a single misaligned chromosome should be identified to be ensheathed by endomembranes (marked by GFP-Sec61β). Acquire live images at 20× with 2.3× intermediate magnification or 60×, 1.40 NA, oil, Plan Apo VC objective; brightfield images are taken at 20× magnification to record the position of the cell on the grid (Figure 1).
*Note: Correlating the selected cell imaged by light microscopy to SBF-SEM is a manual process.*
  4. Following imaging, fix cells in fixation buffer for 1 h. Fixation after imaging must be immediate in order to accurately correlate the light and electron microscopy images. To do this rapidly and to minimize the possibility of mitotic cells detaching from the coverslip, we find that adding the fixative solution directly to the dish while it is still in the microscope is the optimal method. Replace this media-fixative solution with a fresh fixative solution after 1 min.
    **Figure 1.**
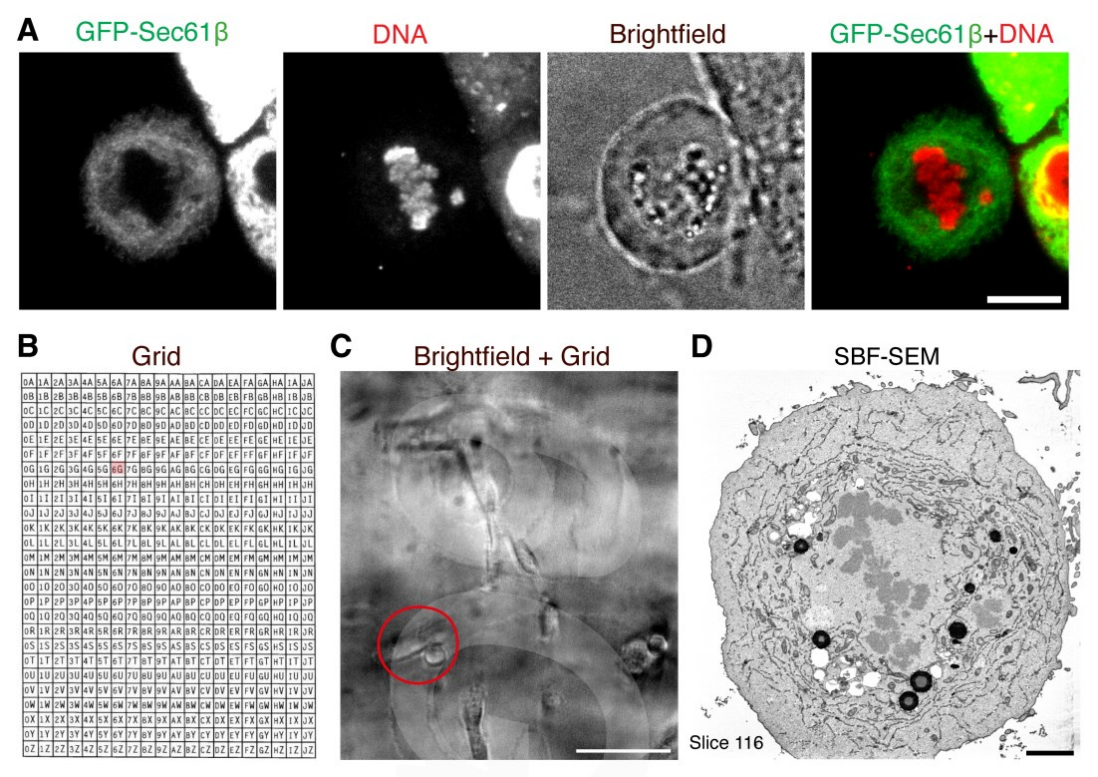
Correlating a cell of interest from light microscopy to electron microscopy. A. Light microscopy imaging of a cell of interest. A mitotic cell expressing GFP-Sec61β is selected at the desired stage of mitosis and with a misaligned chromosome. Scale bar, 10 μm. B. Layout of photoetched grids on MatTek dishes. C. Low magnification image of the position of the cell on the grid (coord 6G can be seen). Scale bar, 100 μm. D. Resulting serial block-face scanning electron microscopy (SBF-SEM) image following correlation. Scale bar, 2 μm.
- **Sample processing**
  1. Perform all processing steps with the cells still in the MatTek dish used for imaging. At all stages, the surface of the dish must be kept covered in order to prevent the sample from drying out. All solution exchanges must be gentle to prevent mitotic cells from detaching from the coverslip.
  2. Following fixation, wash cells three times in PB saline for 5 min each to remove residual aldehydes. **Pause point:** Samples can stay in PB for up to 12 h at 4 °C. This can be useful to collect all the samples before progressing to the next stage.
  3. Postfix, incubate the cells in fresh 2% reduced osmium (equal volumes of 4% aqueous OsO_4_ and 3% potassium ferrocyanide) for 1 h at RT.
  4. During this incubation, prepare a 1% TCH solution for the next step. Always make this solution fresh for each experiment. Add 0.1 g of thiocarbohydrazide to 10 mL of prewarmed dH_2_O and incubate at 60 °C for 1 h. Gently swirl every 5–10 min to facilitate dissolving. It is recommended to filter the solution through a 0.22 μm syringe filter prior to use. TCH acts as a mordant to facilitate heavy metal staining.
  5. Then, wash cells three times for 5 min in PB, removing the excess osmium, at RT.
  6. Incubate cells in prepared TCH for 15 min at RT.
  7. Wash cells three times for 5 min in PB, removing the TCH solution, at RT.
  8. Incubate the cells in an aqueous 2% OsO_4_ solution (not reduced osmium) for 30 min at RT.
  9. Wash the cells three times for 5 min in PB, removing the TCH solution, at RT.
  10. Incubate cells in 1% UA solution in water at 4 °C for 16 h in the dark. Prepare the UA solution from a filtered 20% stock in methanol. Spin down the stock before diluting to remove any precipitate.
  11. Next morning, prepare Walton’s lead aspartate for the next step. To make 10 mL lead aspartate, add 66 mg of lead nitrate to 9 mL of filtered 0.03 M aspartic acid solution and adjust the pH to 4.5 with KOH. Adjust the final volume to 10 mL and the final pH to 5.5. Incubate the lead aspartate at 60 °C for 30 min in a glass vial. The pH adjustment step is tricky and can result in precipitation if an error is made. We adjust the pH in two steps during the preparation of the lead aspartate solution to facilitate dissolution: first to 4.5 with KOH, and then to a final pH of 5.5. The solution is ready to use later if precipitate is not formed. This volume is suitable for 10 samples.
  12. Wash the samples from UA solution following three washes in dH_2_O for 5 min each.
  13. Incubate cells in the previously prepared lead aspartate solution for 30 min at RT.
  14. Remove lead aspartate by washing cells three times in dH_2_O for 5 min each.
  15. Next, dehydrate the samples in a graded series of molecular grade ethanol (30%, 50%, 70%, 90%, and 100%) for 5 min at each concentration.
  16. Now, infiltrate the samples with resin. A hard resin is used when embedding samples for SBF-SEM to minimize beam-induced damage. We use Agar 100 premix kit-Hard. Incubate cells with a mixture of resin:ethanol: first, in a 1:2 proportion for 30 min; second, in a 2:1 proportion for 30 min; and third, full resin for a further 30 min.
  17. Add fresh full resin to fill the MatTek dish (approximately 2 mm).
  18. Polymerize resin to hardness by incubating for 24 h at 60 °C following the manufacturer’s instructions.
  19. Once the resin is cooled to RT, remove the glass coverslip on the MatTek dish by briefly plunging it into liquid nitrogen and using a razor blade to prize the edges of the glass coverslip away from the plastic dish until it detaches, leaving the resin surface and imprinted grid coordinates exposed. The cell of interest is then relocated using the previously acquired brightfield images, and its position is highlighted using a marker pen to aid excision.
  20. Excise the resin block containing the cell of interest using a hacksaw. Trim away excess resin around the grid coordinate using a glass knife on ultramicrotome (EM UC7, Leica Microsystems) to produce a 200 × 200 μm block face. To aid in conductivity around the block face and reduce charging from the electron beam, the block is first painted with silver conductive paste (TAAB) and gold/palladium (80/20%) 60 mm × 0.1 mm thick is subsequently evaporated onto it (Quorum technologies).
- **SBF-SEM**
  SBF-SEM imaging is done using a Gatan 3View system on a FEI quanta 250 ESEM. Load the pin with resin block into the SBF-SEM and relocate the cell of interest using the light microscopy images. Imaging is automated, such that a diamond knife cuts a section, an image is taken, and then the process is repeated. Typically, coarse sections are taken until the cell is in view and then the block is centralized, and higher magnification imaging and fine sectioning is performed. The exact conditions depend on the experiment, but for RPE-1 mitotic cells, imaging at 8.8 nm in *x* and *y* and 60 nm in *z*, in a 4,000 × 4,000 pixel window is sufficient to capture the entire cell and all of the features in which we are interested.
- **Image analysis**
  Large volume EM datasets obtained from SBF-SEM are used for segmentation and subsequent 3D rendering or analysis. The aim is to model the cellular features of the mitotic cell of interest. There are a number of software tools available for segmentation, including Microscopy Image Browser (MIB), AMIRA, ImageJ/Fiji, 3DMOD/IMOD, and Vaa3D. We use a combination of Fiji, MIB, and 3DMOD/IMOD to reconstruct the key features of mitotic cells such as endomembranes and chromosomes.

**FIJI**

1. Import the image sequence into Fiji, using BioFormats to convert the DM3 file to an image stack, or by reading in a series of TIFF images exported by Gatan Digital Micrograph (Schindelin et al., 2012).
2. Ensure that the voxel size is read correctly. If not, correct it using *Image* > *Properties*.
3. It is recommended to convert the 16 bit images into 8 bit to speed up their processing and reduce the final size of the data downstream.
4. SBF-SEM data is inherently in-register because the block is fixed relative to the detector. However, image registration may be required if imaging is interrupted, and the block remounted. Our favorite method is SIFT, to ensure that the stack is in register (*Plugins* > *Registration*).
5. Normalize the contrast throughout the stack by selecting *Process* > *Enhance Contrast*. Change the saturated pixel to 1% and select *Normalize* and *Process All*.
6. OPTIONAL: Pre-process the stack. The goal here is to aid segmentation. Subtraction of a gaussian-blurred version of the stack can reduce uneven density across the image. Additional filters may be used at this stage.
7. Save the adjusted image as a TIFF series using *File* > *Save as Image sequence*.

**MIB**

MIB can be opened through MATLAB or as a standalone program (Belevich et al., 2016). For more information on how to do either, see the MIB website (http://mib.helsinki.fi/).

1. Import the TIFF image stack using Directory Contents.
2. Highlight all the images to be analyzed, right-click, and select *Combine Selected Datasets*.
3. Change the spatial parameters by going to *Dataset* > *Parameters*.
4. If required, it is possible to align or correct the stack for drift by using *Dataset* > *Alignment*, or by choosing *Mode* > *Algorithm* > *Drift correction* (correlate with, color channel, and background). In *Options*, it is possible to align a selected area.
5. Next, start the segmentation process by clicking *Create* in the *Segmentation* panel and select 63 or 255 models, depending on how many objects will be segmented, to start the segmentation.
6. Add a material to the Segmentation panel by clicking the plus button; change the name of the material and it will appear in the column.
7. During segmentation in MIB, pixels can only be selected and assigned to one material at a time. In our case, we determine four materials: chromosomes, mitochondria, ER, and plasma membrane.
8. Each material can be named by double-clicking and selecting the desired color.
9. A variety of tools can then be used for the segmentation of the cellular structures of the mitotic cells.
  - Below the material panel is the segmentation tool drop-down list. The default when you start the program will be the Brush tool, a manual segmentation tool.
  - Use the Brush tool to trace the outline of the object and repeat this over each slice. Press Shift and F to fill the object throughout all the slices.
  - Hold the Ctrl button to turn the brush tool into an eraser and remove any errors.
  - The selected pixels will appear green on the image.
  - Assign the selected pixels to the corrected material in the *Add to* box by choosing the material and pressing Shift and A.
  - MIB offers tools for semiautomatic segmentation:
    i. Interpolation using the brush tool manually: draw on every nth slice and then click I or go to *Selection* > *Interpolation*. Check and correct any errors that have occurred.
    ii. Thresholding takes a user inputted contrast range to select pixels. There are two types: B/W thresholding and the Magic Wand tool.
    1) Select B/W Thresholding from the menu. Alter the range by adjusting the two sliders until a correct selection is made over the entire image.
    2) Click All for B/W thresholding to be applied to the stack of images.
    3) Choose the Magic Wand-Region growing tool and change the variation and radius of the selection tool.
    4) Click on a single pixel in the object—pixels within the range and radius will be selected—and alter the variation and radius until the desired selection is made.
    5) Select 3D in the Selection panel to apply the thresholding to the stack.
    6) Check for any errors and correct them using the brush tool.
10. The chromosomes and plasma membrane of the mitotic cells are segmented using the interpolation tool. Mitochondria were segmented mainly using the thresholding tool. In both cases, the timing was relatively fast for >50 images. However, the ER is segmented manually and is extremely time consuming. We typically render a sub stack using this approach to reduce the time required for manual segmentation. Automated segmentation methods are likely to supplant the manual steps in the next year or so.
11. Save File once the segmentation is finished by *Model* > *Save Model*. We export the models for using IMOD software for 3D reconstruction. Therefore, we export them as Contours (*.mod) and a density factor for points in the XY plane is available depending on the resolution required. In addition, we also need the volume file (*.mrc) through *File* > *Save Image as* > *MRC format* for IMOD, confirming dataset parameters.

**3DMOD/IMOD**

IMOD is a suite of programs to visualize 3D biological image data (Kremer et al., 1996). Instructions to download and launch 3DMOD/IMOD can be found at https://bio3d.colorado.edu/imod/.

1. At the launch window, select Image File (.mrc) and specify the model file (.mod).
2. Load data into the Zap window; all the material and objects are segmented.
3. To visualize the model, select *Image* > *Model View*. This window shows the 3D view of our model (Figure 2A).
4. To mesh the surface follow *Edit* > *Object* > *Meshing* > *Mesh All*. Adjust the settings (brightness, size, color) if they are required (Figure 2B).
5. Save as TIFF by *File* > *Snap TIFF as*. Additionally, a movie/montage can be created.
  **Figure 2.**
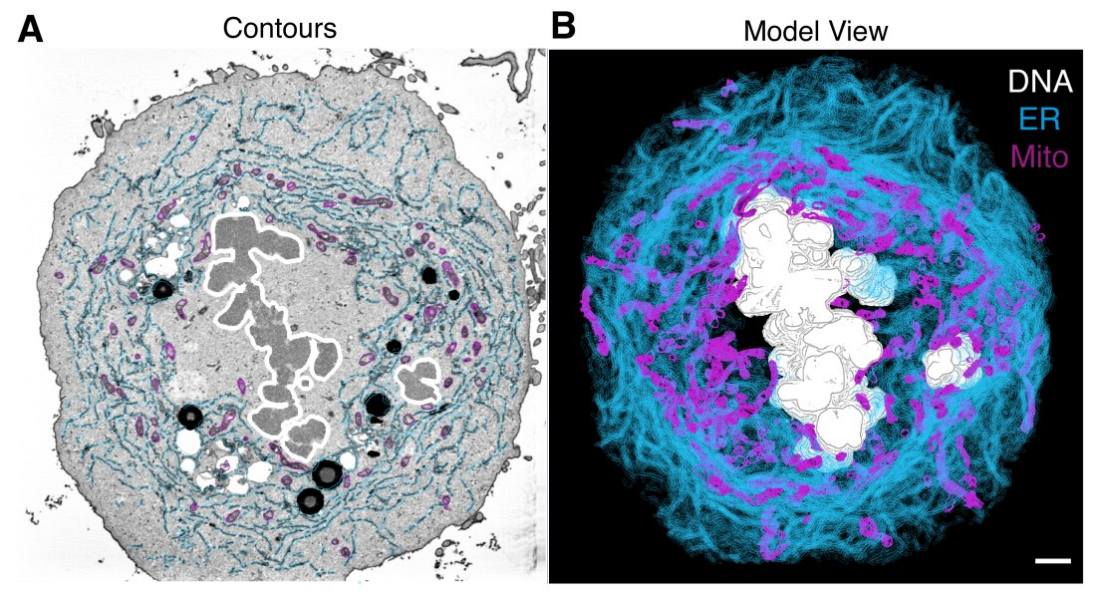
3D model of cellular features. In 3DMOD/IMOD, the 3D model can be viewed in various ways, analyzed, or exported to make figures for publication. Scale bar, 2 μm.

## General notes and troubleshooting

1. Cell density: The density of cells is critical for the accurate reading of grid coordinates. High density can lead to a lack of correlation between cell position and grid coordinates, while low density can result in a reduced number of mitotic cells at the right stage, making the experiment more challenging. A desirable number on the imaging day is around 60% confluency.
2. Imaging mitotic cells: To locate mitotic cells of interest expressing fluorescently tagged proteins, brightfield images are taken at 20× magnification to record the position of the cell relative to the grid coordinates for future reference. The same cell is relocated, and live-cell fluorescence microscopy is performed at 60× magnification (oil objective). Be cautious not to miss the cell of interest when changing objectives. Using 20× with 2.3× intermediate magnification can provide the necessary information depending on the protein of interest and fluorescent protein used.
3. Fixation: Fixation before imaging can be an option, but it should be done with 2% paraformaldehyde in 0.1 M phosphate buffer, pH 7.4, for 1 h. After fixation, wash the samples three times for five minutes each in PB and leave them in PB for imaging. Once imaging is complete, the samples require a second fixation step in 0.5% glutaraldehyde and 0.1% tannic acid in 0.1 M phosphate buffer, pH 7.4, for 1 h before processing with osmium staining. Avoid using glutaraldehyde before imaging, because it causes high autofluorescence, reacting with proteins and peptides to generate visible to near-infrared (IR) emitters.
4. Osmium staining: Make sure the reduced osmium is dissolved correctly before staining the cells. Any precipitates in the dish will not be acceptable for imaging. If the problem persists, it is recommended to filter the solution through a 0.22 μm syringe filter before use. This solution should always be fresh and never reused from previous staining. It is also important to wash the sample properly after fixation before adding the osmium-reduced solution. A hint of aldehyde fixative left in the dish can cause precipitation once the osmium solution is added. When using correlative dishes, ensure all solutions are removed from the coverslip and at least three washes are performed between the fixation and osmium staining steps.
5. Resin polymerization: This is a crucial step because once the coverslip comes off, if a small amount of resin stays on the glass, it is challenging to get the reference grid to identify the correct area. The cells may not have been embedded in the resin properly and slightly stand out of the resin. Additionally, it would make it more difficult to trim the resin as it is either very brittle or still gel-like, causing it to crumble away when trimming is done. The problem may be that the coverslip still contains some solvent and did not mix well enough with the fresh resin to polymerize properly. To avoid these issues, ensure the previous solutions are completely removed before starting the step to embed the samples with resin. Follow the manufacturer’s instructions provided for each resin to determine the time and temperature incubations required for proper polymerization.
6. Analysis tools: There are very helpful online tools, such as tutorials and a mailing list.
  - MIB Tutorial: http://mib.helsinki.fi/tutorials.html
  - IMOD Tutorial: https://www.andrewnoske.com/wiki/IMOD_-_tutorial
https://www.youtube.com/watch?v=Nu7TzloKfWU
https://www.youtube.com/watch?v=enMEEjPDRE0

## Recipes

1. **0.1 M phosphate buffered (PB) saline**
  Dissolve 1.41 g of Na_2_HPO_4_ in 50 mL of H_2_O (0.2 M solution)
  Dissolve 1.2 g of NaH_2_PO_4_ in 50 mL of H_2_O (0.2 M solution)
  Add 40.5 mL of Na_2_HPO_4_ solution to 9.5 mL of NaH_2_PO_4_
  Adjust pH to 7.4 with 10 N KOH solution
  Add 50 mL of H_2_O to make 0.1 M solution
  Storage: 4 °C
2. **Fixation buffer**
  2.5% glutaraldehyde
  2% paraformaldehyde
  0.1% tannic acid
  0.1 M phosphate buffer, pH 7.4
  Storage: Immediate use only
3. **2% reduced osmium**
  4% aqueous OsO_4_
  Dissolve 0.2 g of potassium ferrocyanide power in 10 mL of 0.1 M PB (for 3% solution)
  To make 2% reduced osmium, mix equal volumes of 4% OsO_4_ and 3% potassium ferrocyanide
  Storage: Immediate use only
4. **1% thiocarbohydrazide (TCH) solution**
  Measure 0.1 g of TCH
  10 mL of prewarmed dH_2_O
  Mix the TCH and water and incubate at 60 °C for 1 h
  Gently swirl every 5–10 min to dissolve
  Storage: Immediate use only
*Note: It is recommended to filter the solution through a 0.22 μm syringe filter prior to use.*
5. **1% uranyl acetate (UA)**
  Prepared from a filtered 20% stock in methanol
  Measure 0.5 g of UA
  2.5 mL of methanol
  Mix the UA and the methanol. Dissolve by shaking
  Filter and spin down the 20% stock solution
  To make 1%, take 0.25 mL from 20% in 5 mL of H_2_O
  Storage: 4 °C for up to a month in foil and parafilm lid
*Note: UA precipitates under light.*
6. **Lead aspartate solution**
  Make up 0.03 M aspartic acid stock
  Heat at 60 °C for 60 min to dissolve
  Filter and store at RT
  Add 0.066 g of lead nitrate to 9 mL of 0.03 M aspartic acid stock
  Adjust the pH to 4.5 with 10 N KOH
  Make up to 10 mL of aspartic acid stock
  Adjust the pH to 5.5 with 10 N KOH
  Incubate in a glass vial for 30 min at 60 °C
7. **Agar 100 premix kit**
  One component is in a bottle of 50 mL capacity; second component is in a bottle of 100 mL capacity
  Add the contents of the smaller bottle to the larger bottle
  Add the contents of one of the ampoules of accelerator
  Mix thoroughly and approximately 100 g of resin is ready to use
  Storage: at 4 °C for a few days
*Note: If the unused mixed resin is stored at a reduced temperature before use, the resin must be allowed to reach RT before opening and use.*
